# Highly efficient methods to obtain homogeneous dorsal neural progenitor cells from human and mouse embryonic stem cells and induced pluripotent stem cells

**DOI:** 10.1186/s13287-018-0812-6

**Published:** 2018-03-15

**Authors:** Meixiang Zhang, Justine Ngo, Filomena Pirozzi, Ying-Pu Sun, Anthony Wynshaw-Boris

**Affiliations:** 1 Center for Reproductive Medicine, The First Affiliated Hospital of Zhengzhou University, Zhengzhou, Henan 450052 China; 2 Henan Key Laboratory of Reproduction and Genetics, The First Affiliated Hospital of Zhengzhou University, Zhengzhou, Henan 450052 China; 3 Department of Genetics and Genome Sciences, Case Western Reserve University, Cleveland, OH 44106 USA

**Keywords:** Induced pluripotent stem cells, Embryonic stem cells, Dorsal neural progenitor cells, Neuronal differentiation

## Abstract

**Background:**

Embryonic stem cells (ESCs) and induced pluripotent stem cells (iPSCs) have been widely used to generate cellular models harboring specific disease-related genotypes. Of particular importance are ESC and iPSC applications capable of producing dorsal telencephalic neural progenitor cells (NPCs) that are representative of the cerebral cortex and overcome the challenges of maintaining a homogeneous population of cortical progenitors over several passages in vitro. While previous studies were able to derive NPCs from pluripotent cell types, the fraction of dorsal NPCs in this population is small and decreases over several passages. Here, we present three protocols that are highly efficient in differentiating mouse and human ESCs, as well as human iPSCs, into a homogeneous and stable population of dorsal NPCs. These protocols will be useful for modeling cerebral cortical neurological and neurodegenerative disorders in both mouse and human as well as for high-throughput drug screening for therapeutic development.

**Methods:**

We optimized three different strategies for generating dorsal telencephalic NPCs from mouse and human pluripotent cell types through single or double inhibition of bone morphogenetic protein (BMP) and/or SMAD pathways. Mouse and human pluripotent cells were aggregated to form embryoid bodies in suspension and were treated with dorsomorphin alone (BMP inhibition) or combined with SB431542 (double BMP/SMAD inhibition) during neural induction. Neural rosettes were then selected from plated embryoid bodies to purify the population of dorsal NPCs. We tested the expression of key dorsal NPC markers as well as nonectodermal markers to confirm the efficiency of our three methods in comparison to published and commercial protocols.

**Results:**

Single and double inhibition of BMP and/or SMAD during neural induction led to the efficient differentiation of dorsal NPCs, based on the high percentage of PAX6-positive cells and the NPC gene expression profile. There were no statistically significant differences in the variation of PAX6 and SOX1-positive NPCs between the two human pluripotent cell-derived methods; therefore, both methods are suitable for producing stable dorsal NPCs. When further differentiated into mature neurons, NPCs gave rise to a population of almost exclusively forebrain cortical neurons, confirming the dorsal fate commitment of the progenitors.

**Conclusions:**

The methods described in this study show improvements over previously published studies and are highly efficient at differentiating human and mouse pluripotent cell types into dorsal PAX6-positive NPCs and eventually into forebrain cortical neurons.

**Electronic supplementary material:**

The online version of this article (10.1186/s13287-018-0812-6) contains supplementary material, which is available to authorized users.

## Background

The ability to derive and differentiate human and mouse embryonic stem cells (hESCs and mESCs) into dorsal telencephalic neural progenitor cells (NPCs) has become a useful tool to study early neural development in the forebrain. Moreover, recent advances in reprogramming patient-derived cells into induced pluripotent stem cells (iPSCs) have expanded the possibilities of personalized medicine and understanding the mechanisms underlying neurological diseases with patient-specific mutations [1]. Dorsal NPCs derived from ESCs and iPSCs have been widely used to recapitulate the early processes of neural induction and neuronal differentiation in the forebrain of humans and mice [2–4]. These NPCs derived from pluripotent cell types represent useful models to study the potential defects in neural patterning, proliferation, or differentiation in the human cortex as a result of various developmental neurogenetic and neurodegenerative diseases, such as autism, Alzheimer’s disease, and bipolar disorders [5–7]. Additionally, NPCs have a high proliferative capacity, making them advantageous for high-throughput analyses, such as drug screening for therapeutic development [8].

During early development, the anterior neuroectoderm, comprised of a pseudostratified neuroepithelium, gives rise to the forebrain [9]. Dorsoventral patterning of the forebrain is dictated by the surrounding morphogen gradients and leads to the development of the dorsally positioned telencephalon, which includes the cerebral cortex [9, 10]. The telencephalon in the forebrain gives rise to multipotent, dorsal neural progenitors, which are important for normal corticogenesis [11–13]. Dorsal NPCs are localized in the ventricular zone in the cortex and are marked by the pleiotropic paired box transcription factor PAX6 [14, 15]. In addition to PAX6 expression, SOX1 and Nestin are key markers representative of the neuroepithelium that have been widely used to characterize the NPC population [16–19].

Several neural induction methods have been described for mouse and human ESCs and iPSCs that either involve the initial formation of embryoid bodies (EBs) in suspension cultures or direct neural induction by culturing adherent pluripotent cells in chemically defined media to induce telencephalic forebrain differentiation [17, 20–22]. Various methods describe the use of regionalizing growth factors that drive dorsal NPC and neuronal differentiation, such as agonists for the Wnt, FGF, and Hedgehog pathways and antagonists for BMP and SMAD signaling [3, 17, 20, 23–25]. Although all of these methods demonstrate the presence of prospective dorsal PAX6-positive NPCs, the overall efficiency for generating a large, homogeneous population of these dorsal NPCs is variable [17]. Commercial protocols that require a few days to convert hESCs or hiPSCs into NPCs are also available. However, the commercial protocol utilized in this study was prone to variations between cell lines, with a low percentage of PAX6-positive NPCs observed in some hiPSC lines. Moreover, the population of PAX6-positive NPCs is not maintained and decreases during extended culture, which would limit the use of these cells. Furthermore, many of the protocols described are optimized for established hESC and mESC lines, and more work is needed to improve the optimization of neural induction for hiPSCs as these patient-derived cells may harbor mutations that may potentially affect their neural differentiation efficiency.

In this study, we have optimized strategies for neural induction and generating dorsal NPCs through single BMP and double BMP/SMAD inhibition. The efficiencies in generating dorsal telencephalic NPCs from several mouse ESC and human ESC and iPSC lines using our protocols were compared to commercial and published protocols. For the single BMP inhibition method, we adapted our original protocol designed to induce dorsal NPC differentiation from mESCs to be applicable for hESC and hiPSC neural induction [26]. The double BMP/SMAD inhibition protocol was modified from [23] in order to improve the efficiencies for dorsal NPC differentiation. We demonstrate that our methods are capable of generating a highly homogeneous population of dorsal NPCs that express PAX6/SOX1/Nestin within 14–20 days. Importantly, these human and mouse NPCs are stable, maintain high proliferative capacity throughout multiple passages with minimal spontaneous neuronal differentiation, and, when cultured in the appropriate conditions, generate a population of almost 100% mature cortical neurons. Therefore, the methods described in our study are useful to generate NPCs that can be utilized for mechanistic as well as high-throughput studies, such as drug screening.

## Methods

### Human ESC and iPSC culture and maintenance

De-identified human iPSC lines YH10, BJ4, and 1323–2 were obtained from Dr Bruce Conklin (Gladstone Institutes, described previously in [27]). The H1 ESCs were obtained from Dr Paul Tesar (Case Western Reserve University). Human ESCs and iPSCs were used between passages 20–40, maintained in xeno-free conditions in E8 medium on Vitronectin-coated (Life Technologies) plates, and passaged every 3–5 days to prevent differentiation. Unless otherwise specified, reagents were purchased from Gibco.

### Mouse ESC derivation, cell culture, and maintenance

Mouse ESCs were derived from the inner cell mass of 129/Sv6 *wild-type* blastocysts as described previously [28]. All mESC lines were maintained in LIF (EMD Millipore) conditioned media, which consisted of Iscove’s Modified Dulbecco’s Medium (IMDM) supplemented with 20% Knockout Serum Replacement (KOSR), 1% nonessential amino acids, 1% GlutaMAX, 1% penicillin/streptomycin, and β-mercaptoethanol. Cells were passaged every 3 days with 0.25% trypsin onto 0.1% porcine gelatin-coated plates prior to neural differentiation.

### Generation of human ESC and iPSC-derived NPCs through single BMP inhibition

**Fig. 1:**
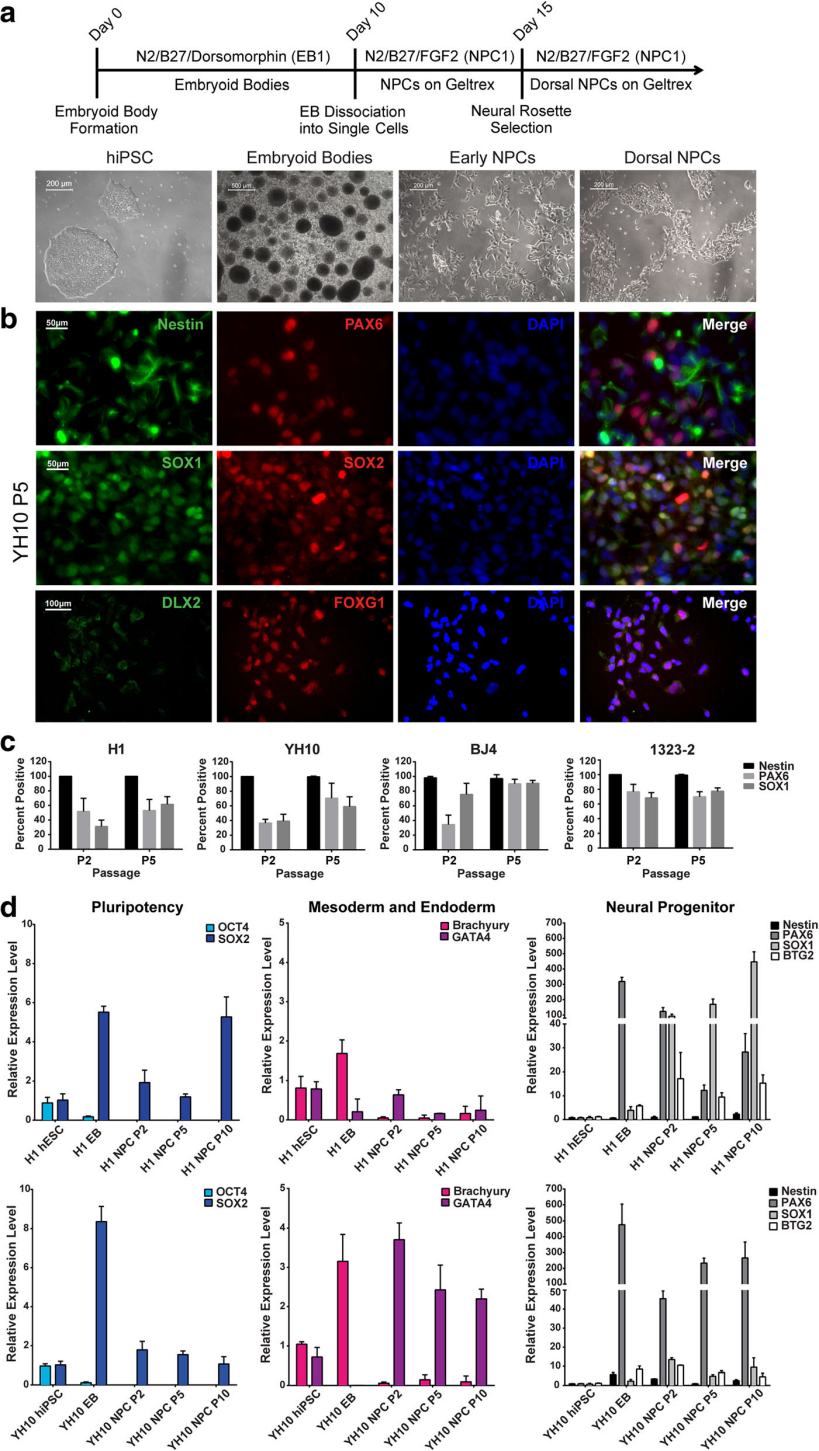
Generation of PAX6-positive dorsal neural progenitor cells (NPCs) from human ESCs and iPSCs via single BMP inhibition. **a** Protocol timeline and representative images of differentiation at different stages. **b** Immunofluorescence staining of passage 5 YH10 NPCs with NPC markers Nestin, PAX6, SOX1, and SOX2, ventral forebrain marker DLX2, and dorsal forebrain marker FOXG1. **c** Quantification of percentage of Nestin, PAX6, and SOX1-positive cells. Total number of cells quantified by DAPI nuclear staining using ImageJ (*n* = 6 independent experiments). **d** Gene expression analysis by qRT-PCR for pluripotency (OCT4, SOX2), mesodermal (Brachyury), endodermal (GATA4), and neural progenitor (Nestin, PAX6, SOX1, and BTG2) markers. Nuclei stained with DAPI. hiPSC human induced pluripotent stem cell, PAX6 paired box protein 6, SOX SRY (sex determining region Y) box, DAPI 4,6′-diamino-2-phenylindole, EB embryoid body, hESC human embryonic stem cell

Human ESCs and iPSCs were detached from Vitronectin-coated plates after treatment with 0.5 mM EDTA and plated at 1 × 10^6^ cells per ultralow-attachment six-well plate to generate embryoid bodies (EBs) cultured in EB1 medium (neurobasal medium, 1% GlutaMAX, 1% penicillin/streptomycin, 1% N2, 2% B27, and 1.25 μM dorsomorphin (Sigma)) for 10 days, with a media change every 3 days. EBs were then dissociated into single cells with Accutase and plated at a density of 3 × 10^5^ cells per well onto Geltrex-coated six-well plates with NPC1 medium (DMEM/F12, 1% GlutaMAX, 1% N2, 2% B27, and 20 ng/ml FGF2 (R&D Systems), freshly added). Neural rosettes were visible within 5 days of culture and were selected using the STEMdiff™ Neural Rosette Selection Reagent (Stem Cell Technologies) according to the manufacturer’s instructions. The process of neural rosette selection was repeated at the following passage to further purify the dorsal NPC population. TrypLE (ThermoFisher) was used to dissociate the NPCs in all subsequent passages, and 10 μM Y-27632 (ROCK inhibitor, Tocris) was added immediately after passaging to enhance cell survival. Fresh NPC1 media were replaced every other day once the cells were adherent. See Fig. 1a for the protocol timeline.

### Generation of human ESC and iPSC-derived NPCs through double BMP/SMAD inhibition

**Fig. 2:**
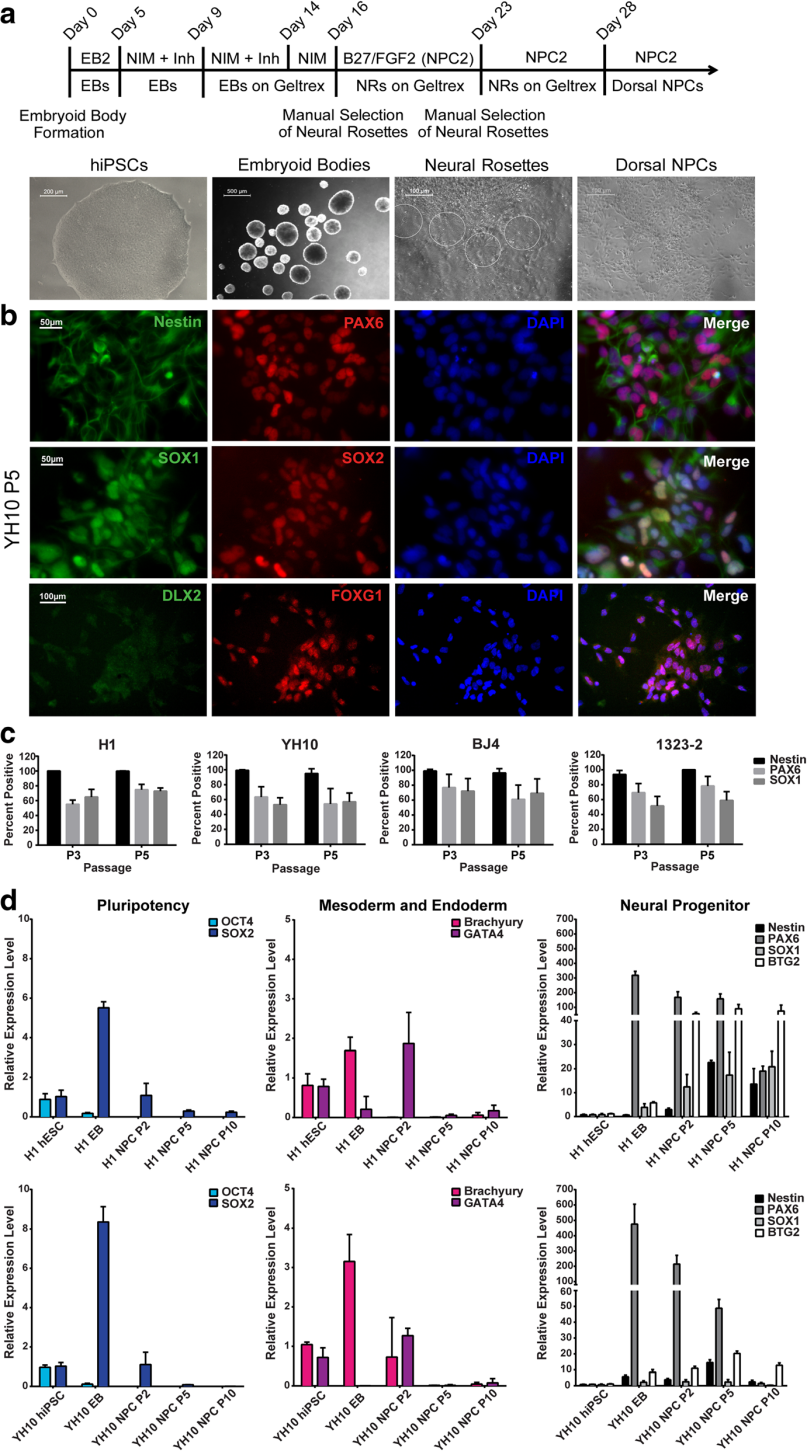
Generation of Pax6-positive dorsal neural progenitor cells (NPCs) from human ESCs and iPSCs via double BMP/SMAD inhibition. **a** Protocol timeline and representative images of differentiation at different stages. **b** Immunofluorescence staining of passage 5 YH10 NPCs with NPC markers Nestin, PAX6, SOX1, and SOX2, ventral forebrain marker DLX2, and dorsal forebrain marker FOXG1. **c** Quantification of percentage of Nestin, PAX6, and SOX1-positive cells. Total number of cells quantified by DAPI nuclear staining using ImageJ (*n* = 6 independent experiments). **d** Gene expression analysis by qRT-PCR for pluripotency (OCT4, SOX2), mesodermal (Brachyury), endodermal (GATA4), and neural progenitor (Nestin, PAX6, SOX1, and BTG2) markers. Nuclei stained with DAPI. EB embryoid body, NIM neural induction medium, Inh inhibitor, FGF2 fibroblast growth factor 2, hiPSC human induced pluripotent stem cell, PAX6 paired box protein 6, SOX SRY (sex determining region Y) box, DAPI 4,6′-diamino-2-phenylindole, hESC human embryonic stem cell

This protocol was slightly modified from [23]. Briefly, hiPSCs and hESCs were cultured in E8 medium on Vitronectin until 80% confluency before NPC differentiation. Cell lines were detached using ReLSR (Stem Cell Technologies), collected, centrifuged, and plated on ultralow-attachment six-well plates to form EBs. The original protocol was followed up to stage IV (NPC production). The following changes were made to optimize dorsal NPC production (see Fig. 2a for the protocol timeline and Additional file 1: Table S2 for a comparison of all protocols): EBs were cultured for 9 days (instead of 8 days) to enhance neuroectoderm differentiation; the first 5 days (instead of 4 days) of EB culture were in EB2 medium (DMEM/F12, 20% KOSR, 1% NEAA, 1% GlutaMAX, β-mercaptoethanol, 5 μM dorsomorphin, and 10 μM SB431542); and the last 4 days of EB culture were in neural induction medium (NIM) (DMEM/F12, 1% NEAA, 1% GlutaMAX, 1% N2, 2% B27, and 10 ng/ml FGF2). Double BMP/SMAD inhibition was extended to day 14 in NIM. EBs were dissociated, plated onto Geltrex-coated plates, and cultured in NIM with 10 ng/ml FGF2 instead of 20 ng/ml, as we found that higher concentrations of FGF2 led to a subsequent loss of PAX6-positive cells using double BMP/SMAD inhibition (data not shown). Adherent EBs were cultured for 7 days and emerging neural rosettes were manually isolated and cultured in NPC2 medium (DMEM/F12, 1% NEAA, 1% GlutaMAX, 1% penicillin/streptomycin, 2% B27, and 10 ng/ml FGF2, freshly added) on Geltrex-coated plates. Neural rosettes isolation was repeated once more on the newly generated NPCs, if necessary. The NPCs were subsequently passaged with TrypLE, and 10 μM Y-27632 was added to minimize apoptosis. Single-cell NPCs were cultured on Geltrex-coated plates in NPC2 medium with FGF2 (10 ng/ml) added fresh every other day.

### Generation of human iPSC-derived NPCs using a commercial protocol

Human iPSCs were dissociated from Vitronectin-coated plates with 0.5 mM EDTA and plated on Geltrex-coated six-well plates. NPCs were generated as a monolayer culture through the usage of a proprietary differentiation supplement that was added according to the manufacturer’s specification and timeline. This protocol did not require manual selection of neural rosettes or population enrichment with microbeads. Nestin and SOX1-positive cells with typical NPC morphology were obtained in 15–20 days.

### Mouse ESC-derived neural progenitor differentiation

**Fig. 3:**
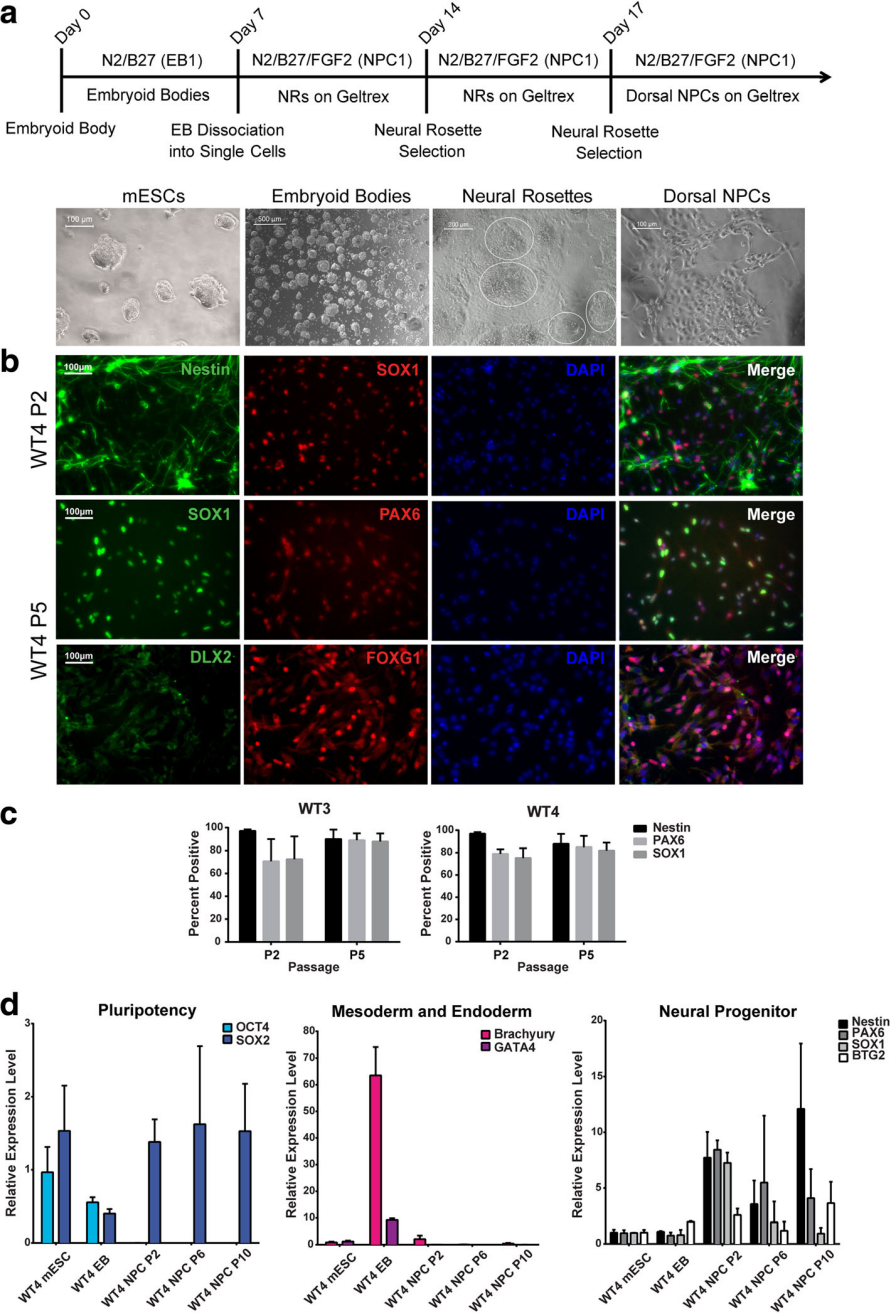
Generation of Pax6-positive neural progenitor cells (NPCs) from mouse ESCs. **a** Timeline for deriving NPCs from mouse ESCs with representative images of differentiation at different stages. **b** Immunofluorescence staining of passage 5 WT4 NPCs with NPC markers Nestin, PAX6, and SOX1, ventral forebrain marker DLX2, and dorsal forebrain marker FOXG1. **c** Quantification of percentage of Nestin, PAX6, and SOX1-positive cells. Total number of cells quantified by DAPI nuclear staining using ImageJ (*n* = 6 independent experiments). **d** Gene expression analysis by qRT-PCR for pluripotency (OCT4, SOX2), mesodermal (Brachyury), endodermal (GATA4), and neural progenitor (Nestin, PAX6, SOX1, and BTG2) markers. Nuclei stained with DAPI. Scale bar 100 μm. EB embryoid body, mESC mouse embryonic stem cell, PAX6 paired box protein 6, SOX SRY (sex determining region Y) box, DAPI 4,6′-diamino-2-phenylindole

Mouse ESCs were removed from a mitotically inactivated MEF feeder layer and cultured on 0.1% porcine gelatin for one passage prior to EB formation. These feeder-free mESCs were detached using 0.25% trypsin and plated in ultralow-attachment six-well plates in EB1 media (neurobasal media, 1% GlutaMAX, 1% penicillin/streptomycin, 1% N2, and 2% B27) without dorsomorphin for 7 days, and EBs were visible by days 2–3. The appearance of neuroepithelium was visible within the EBs at day 7 before the EBs were trypsinized into single cells and plated onto Geltrex-coated plates in NPC1 medium (DMEM/F12, 1% GlutaMAX, 1% N2, 2% B27, and 20 ng/ml FGF2, freshly added). The formation of neural rosettes was visible after 2–3 days and they were cultured for 7 days or until confluency prior to passaging with STEMdiff™ Neural Rosette Selection Reagent. Neural rosette selection was performed during the second passage to further enrich for neuroepithelial NPCs. The dorsal NPCs were expanded using TrypLe and maintained in NPC1 medium on Geltrex-coated plates. The medium was changed every other day with 20 ng/ml FGF2 added freshly. See Fig. 3a for the protocol timeline.

### NPC population doubling time analysis

Two NPC lines per protocol (YH10 and 1323–2) were selected for population doubling time (PDT) analysis. The selected NPCs were cultured from passage 3 to passage 10, seeded at 4 × 10^5^ cells per well in Geltrex-coated six-well plates until 80% confluent. At each passaging, three wells per cell line were then separately dissociated with TrypLE and collected in separate tubes to be counted using the Countess II automated cell counter (ThermoFisher). The PDT was calculated using the equation:

PDT = days in culture × log(2)/ log(final concentration) − log(initial concentration),

where log is a base 2 logarithm. The PDT of the two cell lines was analyzed as a single group to compare the doubling time for each protocol. To assess statistical significance, an unpaired *t* test with Welch’s correction was performed to compare the three protocols as pairs.

### Differentiation of NPCs into mature neurons

In order to determine the actual fate commitment of the generated NPCs, we performed further differentiation in mature neurons. Since the two NPC generation protocols showed no statistically significant difference, we selected the 1323–2 line generated using the double BMP/SMAD inhibition protocol and the WT3 mouse NPCs to further differentiate into mature neurons following the procedure described by Gunhanlar et al. [29]. Briefly, NPCs at passage 7 were dissociated using TrypLE and plated on poly-ornithine/laminin-coated plates and cultured using neural differentiation medium (neurobasal medium, 1% N2 supplement, 2% B27-RA supplement, 1% NEAA, 20 ng/ml BDNF, 20 ng/ml GDNF, 1 μM dibutyryl cyclic adenosine monophosphate (Sigma-Aldrich), 200 μM ascorbic acid (Sigma-Aldrich), 2 μg/ml laminin, and 1% penicillin/streptomycin) for 35 days before immunostaining.

### Immunocytochemistry and imaging analysis

Cells were fixed with 4% formaldehyde for 15 min and permeabilized with 0.5% Triton X-100 in PBS for 10 min. The cells were blocked with 10% donkey serum in PBS for at least 1 h. Primary antibodies against Nestin (Abcam, ab92391), SOX2 (Abcam, ab59776), SOX1 (R&D Systems, AF3369), PAX6 (BioLegend, PRB-278P), Ki67 (CST, 9449S), Dlx2 (Abcam, ab117546), FOXG1 (Abcam, ab18259), MAP2 (Sigma Aldrich M9942), TBR1 (Abcam ab31940), BRN2 (Cell Signaling 12,137), NeuN (Abcam ab104225), SOX9 (R&D Systems AF3075), GLT1 (Novus Biologicals NBP1–20136), and GFAP (Dako Z0334) were diluted in blocking buffer and incubated overnight at 4 °C. Following several washes, the cells were incubated with the appropriate donkey anti-rabbit IgG, anti-mouse IgG, or anti-goat conjugated with Alexa Fluor 488, Alexa Fluor 555, or Alexa Fluor 647 secondary antibodies (Life Technologies) for 1 h at room temperature. Cells were counterstained with DAPI and visualized with the Leica DM6000 inverted microscope. Images were acquired using the Q-Imaging Retiga Xi Firewire High-Speed, 12-bit cooled CCD camera and Volocity software. Staining was quantified using ImageJ software and represented as a portion of total nuclei. On average, 2.5 × 10^3^ cells were quantified from each experiment. Results are shown with the standard deviation of the mean of three independent experiments.

### Gene expression analysis by quantitative RT-PCR

Total RNA was isolated from cells using the RNeasy Plus Mini Kit (Qiagen). The RNA concentration was measured using Nanodrop and 100 ng of each sample was reverse transcribed using Superscript IV VILO Master Mix (Invitrogen) following the manufacturer’s instructions. For real-time detection of mRNA expression, 0.5 μl of 1:3 diluted first-strand cDNA was used as a template for PCR amplification using POWER Up SYBR Green PCR Master Mix (Life Technologies). qRT-PCR was performed with Life Technologies QuantStudio™ 12 K Flex Real-Time PCR System. Primer pairs (Additional file 2: Table S1) were selected from PrimerBank (https://pga.mgh.harvard.edu/primerbank/) or custom designed using Primer-BLAST (https://www.ncbi.nlm.nih.gov/tools/primer-blast/) and tested for equivalent efficiency. Each reaction was carried out in triplicate and fold changes were calculated using the comparative Ct method as described previously [30]. The resulting Ct values were normalized to either β-actin in hESCs and hiPSCs or GAPDH in mESCs. Results are shown ± SD of the mean of three independent experiments, unless otherwise stated.

### Statistical analysis

Statistical analysis to assess differences in dorsal NPC generation among the three protocols was performed using Microsoft Excel and GraphPad Prism v6.0. The mean values are shown with the SD. Statistical significance was calculated using Student’s unpaired *t* test and *F* test for variance (Additional file 3: Table S3). *P* ≤ 0.05 was considered statistically significant. To assess statistical significance in the PDT results, an unpaired *t* test with Welch’s correction was performed to compare the BMP inhibition, the BMP/SMAD inhibition, and the commercial protocols.

## Results

### BMP inhibition promotes the differentiation of human ESCs and iPSCs into telencephalic dorsal NPCs

Several commercial products are available to induce neural differentiation, and we tried one of these methods, according to the manufacturer’s instructions, to generate human iPSC-derived dorsal telencephalic NPCs (Additional file 4: Figure S1A). To verify the presence of dorsal NPCs, we performed immunocytochemical staining for NPC markers (Nestin, PAX6, and SOX1) and SOX2, which is initially expressed in iPSCs as a pluripotency marker but is also required during neural induction [31–33]. The NPCs displayed positive Nestin, SOX1, and SOX2 staining; however, the key dorsal NPC marker PAX6 was absent (Additional file 4: Figure S1B), suggesting that the NPCs generated were not the dorsal subtype. In addition, we performed qRT-PCR to determine the expression levels of several NPC markers and observed low PAX6 expression concordant with our immunostaining (Additional file 4: Figure S1C).

Due to the proprietary nature of the formulations of these commercial reagents, our ability to optimize differentiation was limited. Therefore, we adapted a protocol for differentiating mouse ESCs into dorsal NPCs [26] for human ESCs (H1) and iPSCs (YH10, BJ4, 1323–2). All of the undifferentiated human cell lines were maintained on Vitronectin-coated plates with E8 media and expressed the key pluripotency markers OCT4, SOX2, KLF4, LIN28, and NANOG prior to neural induction (data not shown). To induce NPC differentiation, the hESC and hiPSC colonies were dissociated into single cells and allowed to aggregate into EBs in EB1 medium containing dorsomorphin (Fig. 1a). Dorsomorphin has a potent inhibitory effect on the BMP type I receptors ALK2, ALK3, and ALK6, which has been shown to improve neural differentiation efficiency to drive dorsal specification [20, 23, 24, 34]. The EBs were cultured in suspension for 10 days to allow the formation of self-organized neuroepithelium that will give rise to dorsal NPCs. The EBs were then dissociated into single cells and cultured as an adherent monolayer in the presence of FGF2 without dorsomorphin. A small portion of the neuroepithelial cells organized into neural rosettes, which we were able to select using the STEMdiff™ Neural Rosette Selection Reagent. Based on immunocytochemical staining and qRT-PCR analyses, the hESC and hiPSC-derived NPCs express the dorsal neural progenitor markers Nestin, PAX6, SOX1, and SOX2 (Fig. 1b–d, Additional file 5: Figure S2). Dorsal forebrain identification of these NPCs was further confirmed with the expression of the dorsal marker FOXG1, and the absence of the ventral marker DLX2, in the nucleus (Fig. 1b). We observed a high percentage of Nestin-positive cells in all of the NPC lines derived from human ESC and iPSC types during the early stages of differentiation at passage 2 (Fig. 1c), followed by increasing PAX6 and SOX1 expression at passage 5. At passage 2, the percentages of PAX6-positive and SOX1-positive cells varied among the hESC and hiPSC-derived lines, suggesting that there are cell line-specific differences during the early stages of NPC differentiation. However, by passage 5 the average percentages of hiPSC-derived NPCs expressing Nestin, PAX6, and SOX1 were 98.77% ± 3.02%, 80.51% ± 14.37%, and 72.08% ± 16.42%, respectively. We observed similar efficiencies for the hESC-derived NPC line, suggesting that we were able to obtain highly homogeneous populations of dorsal NPCs from all of the human-derived cell lines.

To determine the neural differentiation efficiency for each cell line, we quantified the expression levels of several pluripotency (OCT4, SOX2), mesodermal (Brachyury), endodermal (GATA4), and dorsal NPC (Nestin, PAX6, SOX1, BTG2) genes using qRT-PCR throughout the process of neural differentiation (Fig. 1d, Additional file 5: Figure S2A). We confirmed the downregulation of OCT4 in the EBs by day 4 of differentiation in the hESC and hiPSC-derived lines, and it was undetectable in the NPCs (Fig. 1d, Additional file 5: Figure S2A). Although the expression of SOX2 is associated with pluripotency, it has also been shown to be involved in NPC differentiation [31, 33]. We observed increased SOX2 levels in the EBs followed by a decreased and steady expression pattern after the early differentiation stage (Fig. 1d, Additional file 5: Figure S2A). This expression profile is in accordance with the proliferative and multipotent nature of NPCs and with the cell populations expected to be present at the different time points, as demonstrated by previous studies [32, 35]. The expression of Brachyury was present in the EBs, and after neural rosette selection Brachyury was significantly decreased in the NPCs (Fig. 1d, Additional file 5: Figure S2A). GATA4 expression persisted in the NPCs derived from the hESCs and hiPSCs (Fig. 1d, Additional file 5: Figure S2A), but more so from the hiPSC lines. We observed decreased GATA4 in NPCs generated from the double BMP/SMAD protocol described in the next section, suggesting that inhibition of BMP alone may not be sufficient to fully suppress endodermal GATA4. However, the expression of GATA4 did not appear to interfere with neural differentiation as we observed increases in the NPC-associated genes Nestin, PAX6, SOX1, and BTG2 by passage 5 that persisted at high levels through passage 10 in our analysis (Fig. 1d, Additional file 5: Figure S2A). The overall pattern of Nestin, PAX6, and SOX1 mRNA expression was consistent with previous studies [36] despite our observation of higher initial Nestin protein expression via immunocytochemical staining during the earlier stages of NPC differentiation (Fig. 1c).

### Double BMP/SMAD inhibition promotes dorsal NPCs derived from human ESCs and iPSCs

A previously published method to generate NPCs was tested [23] and slightly modified in order to obtain a higher percentage of PAX6-positive cells. The same hESC and hiPSC lines mentioned earlier were used for NPC differentiation. Briefly, the cells were cultured as EBs in EB2 medium, supplemented with two SMAD inhibitors: dorsomorphin (BMP receptor inhibitor) and SB431542 (TGF-β inhibitor). SB431542 blocks phosphorylation of the ALK4, ALK5, and ALK7 receptors, and the synergistic effects of dorsomorphin and SB431542 selectively inhibit the TGF-β/Activin/NODAL signaling pathway to promote neuroectodermal differentiation [37]. Changes to the original protocol are described in Methods, and the protocol timeline is displayed in Fig. 2a. The similarities and differences among the four described protocols are presented in Additional file 1: Table S2. The EBs were seeded and cultured on Geltrex-coated plates, which allowed the cells to organize into a monolayer of neural rosettes (Fig. 2a). To test for neural differentiation efficiency, we performed immunostaining for known NPC markers, and by passage 5 we observed Nestin-positive (97.17% ± 5.17%), PAX6-positive (64.52% ± 19.79%), and SOX-positive (63.62% ± 16.51%) cells among the hiPSC-derived NPC lines (Fig. 2b, c). The percentage of PAX6-positive cells indicates that the population of NPCs was highly homogeneous with a major component of dorsal NPCs. Furthermore, these NPCs express FOXG1, but not DLX2, in the nucleus, which further supports their dorsal forebrain identity (Fig. 2b). In addition, we performed gene expression qRT-PCR analysis on the human ESCs and iPSCs, EBs, and NPCs at passages 2, 5, and 10 (Fig. 2d, Additional file 5: Figure S2B). Similar to our BMP inhibition protocol, we observed downregulation of OCT4 by day 4 in the EBs and total repression by passage 2 in the derived NPCs. Transient expression of Brachyury and GATA4 was present in the EBs but became undetectable in the NPCs by passage 5 for all cell lines. Concurrently, the expression of neuroectodermal genes increased throughout EB formation and throughout NPC differentiation and was comparable for the hESC and hiPSC lines, suggesting that this method, in addition to our single BMP inhibition method, is robust for generating dorsal NPCs (Fig. 2d, Additional file 5: Figure S2B).

### Mouse ESCs readily differentiate into Pax6-positive dorsal NPCs

Mouse ESCs were derived from 129/Sv6 *wild-type* blastocysts, and the expression of pluripotency markers were confirmed (Additional file 6: Figure S3) prior to dorsal neural progenitor differentiation. Adherent mESC colonies were dissociated into single cells and allowed to aggregate to form EBs over a period of 7 days in EB1 medium without dorsomorphin (Fig. 3a). The EBs were then dissociated and plated as a monolayer to allow neural rosettes to organize. The neural rosettes were isolated and enriched over two passages using the STEMdiff™ Neural Rosette Selection Reagent. We confirmed the efficiency of generating dorsal NPCs through the positive expression of the neural progenitor markers (Nestin, PAX6, SOX1) and a dorsal forebrain marker (FOXG1), as well as the absence of nuclear DLX2 expression, which is a ventral forebrain marker (Fig. 3b, c). Similar to the results described for the human-derived NPCs, we confirmed the downregulation of pluripotency and nonectodermal genes during the multiple stages of neural induction along with the upregulation of neuroectodermal genes in the NPCs by qRT-PCR (Fig. 3d). Overall, this method is effective in generating dorsal PAX6-positive NPCs from mouse ESC lines.

### Dorsal NPCs generated from single and double BMP inhibition protocols have similar NPC marker signature and proliferation

In order to establish whether the two described protocols for human cell lines are significantly different, we compared the results of our immunofluorescence staining from YH10, BJ4, and 1323–2-derived NPC lines at passage 5 after differentiation (Figs. 1c and 2c, Additional file 1: Table S2 and Additional file 3: Table S3). Overall, there was no significant difference in the expression of NPC markers (*F* test for variance), except for cell line BJ4 which showed higher PAX6 and SOX1 expression levels using the BMP inhibition protocol (Fig. 1c). These differences may be due to cell line-specific characteristics, but our results suggest that both of the described protocols are suitable for human NPC derivation.

A key feature of NPCs is their ability to rapidly and consistently proliferate. The human and mouse cell lines from all of the described NPC differentiation protocols showed similar expression levels of the proliferative marker Ki67 (Additional file 7: Figure S4A–C). Depending on the NPC subtype, the average population doubling time (PDT) can range from 1.2 to 2.1 days. The NPCs generated with the BMP inhibition and the double BMP/SMAD inhibition protocols displayed an average PDT of 1.61 and 1.65 days, respectively (Additional file 7: Figure S4D), and this difference was not statistically significant. In contrast to these results, the NPCs generated using the commercial protocol showed a significantly different PDT (2.29 days, *p* < 0.0001), mostly due to a delayed doubling time in later passages (Additional file 7: Figure S4D).

### Dorsal NPCs differentiate into a highly homogeneous population of forebrain cortical neurons

**Fig. 4:**
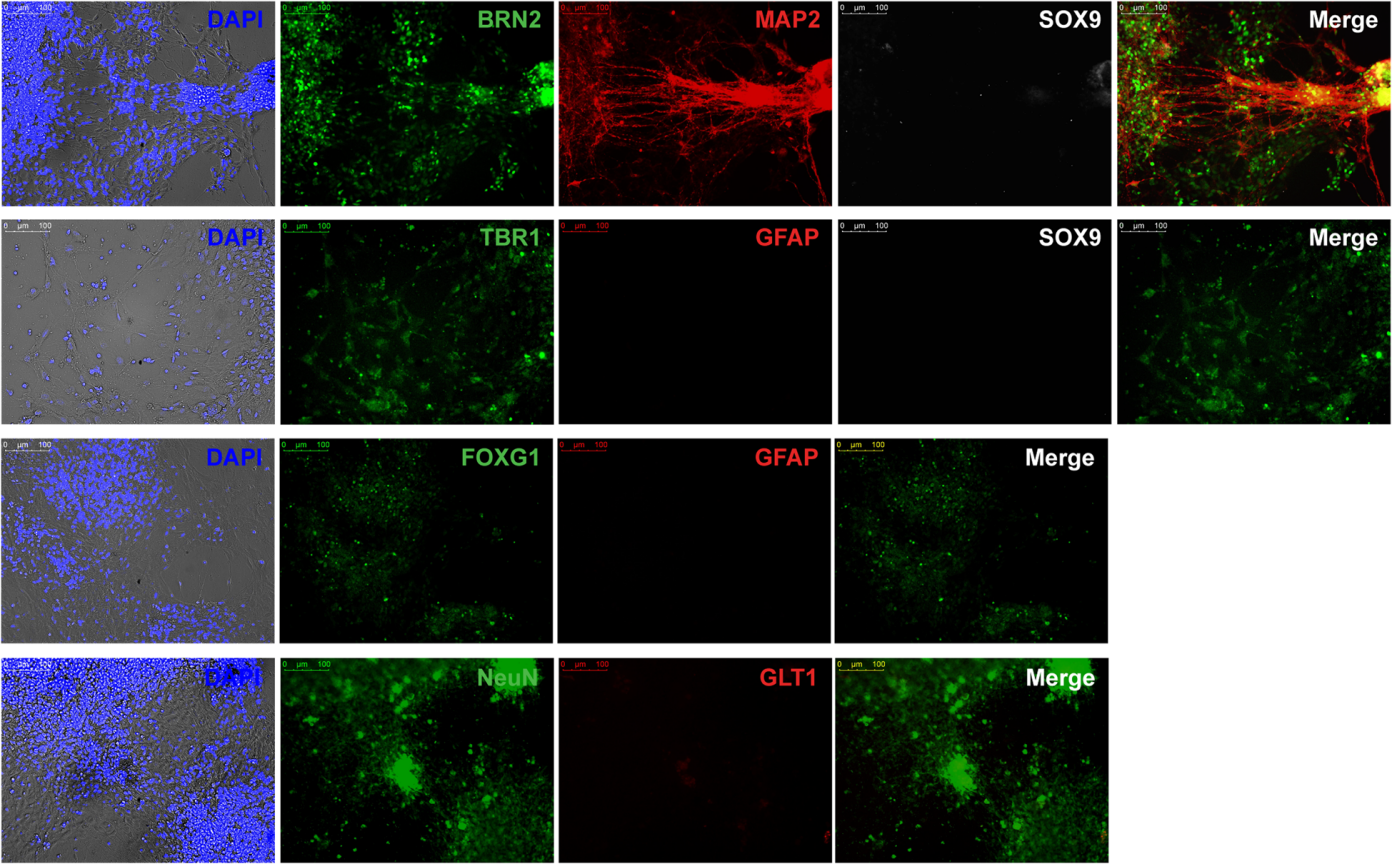
Human NPCs differentiate into mature neurons. Immunofluorescence staining of differentiated neurons derived from human dorsal NPCs (1323–2 line, day 35 after differentiation) for mature cortical neuronal markers expressed in the nucleus (BRN2, TBR1, NeuN) and cytoplasm (MAP2), glial markers (SOX9, GFAP, GLT1), and dorsal forebrain marker (FOXG1). Nuclei stained with DAPI, shown as an overlay over brightfield images. The merge is an overlay of the neuronal and glial markers. Scale bar 100 μm. DAPI 4,6′-diamino-2-phenylindole

The expression levels of dorsal NPCs markers, such as PAX6, SOX1, and FOXG1, confirmed the forebrain identify of our NPCs. However, to demonstrate that the NPCs behaved liked functional progenitors, we differentiated the NPCs into mature cortical neurons. We adopted a recently published method [29], in which a heterogeneous population of NPCs was cultured in neuronal inducing conditions for 4–10 weeks, leading to electrophysiologically mature neuronal networks [29]. We performed the differentiation protocol using human 1323–2 NPCs derived using the BMP/SMAD inhibition protocol (Fig. 4) and mouse WT3 NPCs (data not shown), and cultured them for 35 days before performing immunostaining. To characterize the population of mature neurons obtained, we used a forebrain telencephalic marker, FOXG1; a superficial cortical layer (II–IV) marker, BRN2; a deep cortical layer (V and VI) marker, TBR1; a neuronal nuclear marker, NeuN; a dendritic marker, MAP2; and astrocyte markers GFAP, SOX9, and GLT1. The human NPCs successfully differentiated into a population of almost 100% cortical forebrain neurons, as confirmed by the presence of staining for the cortical markers and the absence of staining for all three astrocyte markers (Fig. 4). The mouse NPCs, although plated at a lower density, were also able to differentiate into neurons that were TBR1-positive while lacking expression of GFAP and SOX9 (Additional file 8: Figure S5).

## Discussion

Our previous attempts to use commercially available reagents to induce NPC differentiation failed to effectively produce PAX6-positive human dorsal NPCs, the progenitor subtype that is found in the cerebral cortex, despite the presence of cells expressing Nestin, SOX1, and SOX2. Dorsal NPCs may require additional signaling or growth factors that are not present in the proprietary formulations of commercial neural induction media. Previously published methods [17, 20, 22, 23] were also able to obtain proliferative NPCs; however, these populations were heterogeneous, with variable PAX6 expression that was not quantified over long-term passaging. In order to generate stable PAX6-positive NPCs, we modified a neural differentiation protocol originally developed for mESC-derived NPCs [26], and adapted it for human pluripotent cell types. We also optimized a method described in [23] to produce more PAX6-positive dorsal NPCs. For each method described, we monitored the expression of dorsal NPC markers for at least 10 passages to confirm that the NPCs produced are maintained throughout extended passages.

All three methods described in this study are efficient at producing highly homogeneous populations of PAX6-positive dorsal NPCs derived from mouse and human ESCs and iPSCs within 2 weeks of neural induction. The two neural differentiation protocols for human cell types displayed similar neural induction efficiencies. Importantly, the derived NPCs displayed fast and stable proliferation, and minimal spontaneous neuronal differentiation over many passages. Although none of the protocols produce completely homogeneous PAX6-positive cells, the protocols described in this study are an improvement over current methods. Should there be a need for obtaining pure PAX6-positive NPCs, additional purification using a PAX6 reporter system followed by FACS should be sufficient to eliminate the residual nondorsal NPCs. In addition, we confirmed that the human and mouse NPCs generated from our protocols are representative of the dorsal forebrain based on the expression of the dorsal marker FOXG1 and the absence of nuclear expression of the ventral marker DLX2. Moreover, utilizing EB formation rather than direct monolayer neural induction methods may be more beneficial for studies that require a large population of dorsal NPCs to be generated in a shorter period of time, as demonstrated by Chandrasekaran et al. [38]. Choosing between single BMP or double BMP/SMAD inhibition to induce neural differentiation may depend on whether the identification of neural rosettes is required. We found that the double BMP/SMAD inhibition in human pluripotent cells was better suited for applications that require visual neural rosette observation, presumably because the inhibition of these factors provided the necessary signals for longer culture maintenance.

We compared the neural differentiation efficiencies of single BMP inhibition and double BMP/SMAD inhibition among the human cell lines, and observed some variability in gene expression among the different hiPSC lines. Although neural differentiation of hESCs and hiPSCs was similarly efficient based on our immunostaining analysis, the hESC line displayed higher transcriptional levels of NPC markers. We speculate that the lower level of NPC markers in the hiPSC lines may be due to the retention of an “epigenetic memory” that is not completely lost during the process of cellular reprogramming [39–41]. In addition, differences in the method of reprogramming and of the genetic background among these commercially available hiPSC lines could further contribute to the variability in neural differentiation efficiency [42–44]. This overall variability may be relatively minor since we were able to generate a homogeneous population of PAX6-positive dorsal NPCs and statistical analysis of this variability was not significant.

We further confirmed that the NPCs generated by our methods are capable of differentiating into forebrain cortical mature neurons using a differentiation protocol developed by Gunhanlar et al. [29]. In their original work, the final neuronal network was composed of 59.5% mature neurons versus 40.5% astrocytes, while our final neuronal population showed cells that were only positive for forebrain cortical markers such as FOXG1, NeuN, BRN2, TBR1, and MAP2, suggesting that our initial population of NPCs was indeed enriched in dorsal forebrain committed cells and lacked a ventral component.

## Conclusions

Our three protocols are efficient, reliable, and robust methods to generate homogeneous dorsal NPC populations from both mouse and human ESC and hiPSC lines compared to previously published studies and commercial neural induction products. Here, we have optimized available protocols in order to produce dorsal NPCs that are stable over several passages and useful for potential high-throughput screening studies. These NPCs are also capable of differentiating into a highly homogeneous population of forebrain cortical neurons.

## Additional files

Additional file 1: is **Table S2** presenting a comparison of the four described protocols. Comparison of the two newly described protocols (BMP inhibition and Double BMP/SMAD inhibition) with the previously published double SMAD protocol (Original Mak et al.) and the commercial protocol tested. #For detailed recipes of media refer to the methods section. *The commercial protocol did not produce any PAX6+ cells in our hands, however the manufacturer's manual specifies that this population of cells was present and averaged 15-50%. iMEF: inactivated Mouse Embryonic Fibroblasts; hES: human Embryonic Stem cell medium; NA: Not available. (DOCX 15 kb)

Additional file 2: is **Table S1** presenting primers used for qRT-PCR represented in Figs. 1–3 and Additional file 5: Figure S2. (DOCX 15 kb)

Additional file 3: is **Table S3** presenting testing for statistically significant differences in PAX6 and SOX1 IHC staining between single BMP and double BMP/SMAD neural induction protocols. Significance testing for differences in NPC marker expression for each human cell line in the single BMP and double BMP/SMAD inhibition protocols. Student's unpaired T-test and F test for variance were performed. P-values are shown comparing the ICC quantification in Fig. 1c and Fig. 2c; *p*-values <0.05 were considered significant. (DOCX 14 kb)

Additional file 4: is **Figure S1** showing differentiation of hiPSCs into NPCs using commercial reagents. Two hiPSC lines (YH10 and 1323–2) were prepared and differentiated according to the manufacturer’s instructions. (**A**) Representative images of 1323–2 line taken on days 1, 3, 5, 7, and 8 of NPC differentiation showing morphology of hiPSC colonies transitioning into NPCs. (**B**) NPCs derived from 1323–2 fixed and stained with Nestin, PAX6, SOX1, and SOX2 at passage 5. Notably, these NPCs fail to express dorsal marker PAX6 (**B**, **C**). (**C**) qRT-PCR analysis of pluripotency (OCT4, SOX2), mesodermal (Brachyury), endodermal (GATA4), and neural progenitor (Nestin, PAX6, and SOX1) genes in YH10 and 1323–2 hiPSC-derived NPC lines. Nuclei stained with DAPI. Scale bar 100 μm. (TIFF 9638 kb)

Additional file 5: is **Figure S2** (related to Figs. 1 and 2) showing comparison of gene expression profiles during NPC differentiation among the single BMP and double BMP/SMAD inhibition protocols. (**A**, **B**) qRT-PCR of pluripotency (OCT4, SOX2), mesodermal (Brachyury), endodermal (GATA4), and neural progenitor (Nestin, PAX6, and SOX1) genes in YH10 and 1323–2 lines generated via (**A**) BMP inhibition and (**B**) double SMAD inhibition. (TIFF 2381 kb)

Additional file 6: is **Figure S3** showing mESCs express pluripotency genes. Immunofluorescence staining of mESCs using pluripotency markers SOX2 and LIN28. Nuclei stained with DAPI. Scale bar 100 μm. (TIFF 3029 kb)

Additional file 7: is **Figure S4** showing human and mouse NPCs are highly proliferative. Immunofluorescence staining of (**A**, **B**) human and (**C**) mouse dorsal NPCs for proliferation marker Ki67. NPCs generated via (**A**) single BMP inhibition, (**B**) double SMAD inhibition, and (**C**) mouse protocols are positive for Ki67. (**D**) Doubling time of NPCs derived from our protocols are consistent, while doubling time of NPCs derived from commercial methods are more variable. Nuclei stained with DAPI. Scale bar 100 μm. Unpaired *t* test with Welch’s correction was performed to compare BMP inhibition, BMP/SMAD inhibition, and commercial protocols. ***p < 0.0001. (TIFF 7781 kb)

Additional file 8: is **Figure S5** showing mouse NPCs differentiate into mature forebrain cortical neurons. Immunocytofluorescence staining of differentiated neurons derived from mouse dorsal NPCs for mature neuronal (TBR1) and glial (GFAP and SOX9) markers. Nuclei stained with DAPI. Scale bar 100 μm. (TIFF 2348 kb)

## Abbreviations

BMP: Bone morphogenetic protein
DAPI: 4,6′-Diamino-2-phenylindole
DMEM/F12: Dulbecco’s Modified Eagle Medium: Nutrient Mixture F-12
EB: Embryoid body
ESC: Embryonic stem cell
FGF2: Fibroblast growth factor 2
iPSC: Induced pluripotent stem cell
KOSR: Knockout serum replacement
NEAA: Nonessential amino acid
NIM: Neural induction medium
NPC: Neural progenitor cell
PAX6: Paired box protein 6
SOX2: SRY (sex determining region Y) box 2
TGF-β: Transforming growth factor beta
