# Supplementary material for: Highly efficient methods to obtain homogeneous dorsal neural progenitor cells from human and mouse embryonic stem cells and induced pluripotent stem cells
Source: Stem Cell Res Ther. 2018 Mar 15;9:67. doi: 10.1186/s13287-018-0812-6 (PMC5856210; doi:10.1186/s13287-018-0812-6)
Supplement: Supplementary file 1 — is Table S2 presenting a comparison of the four described protocols. Comparison of the two newly described protocols (BMP inhibition and Double BMP/SMAD inhibition) with the previously published double SMAD protocol (Original Mak et al.) and the commercial protocol tested. #For detailed recipes of media refer to the methods section. *The commercial protocol did not produce any PAX6+ cells in our hands, however the manufacturer's manual specifies that this population of cells was present and averaged 15-50%. iMEF: inactivated Mouse Embryonic Fibroblasts; hES: human Embryonic Stem cell medium; NA: Not available. (DOCX 15 kb) [file 13287_2018_812_MOESM1_ESM.docx]

**Table S2:** Comparison of the four described protocols.

|  | BMP inhibition | Double BMP/SMAD inhibition | Original Mak et al[23] protocol | Commercial protocol |
| --- | --- | --- | --- | --- |
| iPSC culturing conditions | Vitronectin and E8 Medium | Vitronectin and E8 Medium | iMEFs and hES medium | Vitronectin and E8 Medium |
| iPSC dissociation | EDTA 0.5mM | ReLSR® | Collagenase IV 1mg/ml | StemPro® Accutase® |
| EBs vs. monolayer | EBs | EBs | EBs | Monolayer |
| Inhibitors | Dorsomorphin | Dorsomorphin, SB431542 | Dorsomorphin, SB431542 | Unknown |
| Adhesion Matrix | Geltrex® | Geltrex® | Geltrex® | Geltrex® LDEV-Free |
| Final NPC Medium^#^ | DMEM/F12, N2, B27, FGF (20ng/ml)  (NPC1 medium) | DMEM/F12, N2, B27, FGF (10ng/ml)  (NPC2 medium) | Neurobasal, B27, FGF2 (20ng/ml) | Commercial induction medium |
| Selection of Neural Rosettes | Yes (2x) | Yes (1-2x) | Yes (2x) | No |
| Microbeads Population Enrichment | No | No | Yes | No |
| Total Length of Protocol (days) | 15-20 | 16-23 | 18-22 | 8-14 |
| % of PAX6^+^ Cells | 80.51%±14.37% | 64.52%±19.79% | NA | 0% ^*^ |

Comparison of the two newly described protocols (BMP inhibition and Double BMP/SMAD inhibition) with the previously published double SMAD protocol (Original Mak et al.) and the commercial protocol tested. #For detailed recipes of media refer to the methods section. *The commercial protocol did not produce any PAX6+ cells in our hands, however the manufacturer’s manual specifies that this population of cells was present and averaged 15-50%. iMEF: inactivated Mouse Embryonic Fibroblasts; hES: human Embryonic Stem cell medium; NA: Not available
