# Supplementary material for: Highly efficient methods to obtain homogeneous dorsal neural progenitor cells from human and mouse embryonic stem cells and induced pluripotent stem cells
Source: Stem Cell Res Ther. 2018 Mar 15;9:67. doi: 10.1186/s13287-018-0812-6 (PMC5856210; doi:10.1186/s13287-018-0812-6)
Supplement: Supplementary file 2 — is Table S1 presenting primers used for qRT-PCR represented in Figs. 1–3 and Additional file 5: Figure S2. (DOCX 15 kb) [file 13287_2018_812_MOESM2_ESM.docx]

**Table S1** Primers used for qRT-PCR represented in Figs 1, 2, 3 and Additional file 5 Figure S2.

| SOX1 FW | GAGATTCATCTCAGGATTGAGATTCTA |
| --- | --- |
| SOX1 RV | GGCCTACTGTAATCTTTTCTCCAC |
| B-ACTIN FW | CTGAACCCCAAGGCCAAC |
| B-ACTIN RV | TAGCACAGCCTGGATAGCAA |
| OCT4 FW | TGGGCTCGAGAAGGATGTG |
| OCT4 RV | GCATAGTCGCTGCTTGATCG |
| NESTIN FW | TGCGGGCTACTGAAAAGTTC |
| NESTIN RV | AGGCTGAGGGACATCTTGAG |
| BRACHYURY FW | AGGTACCCAACCCTGAGGA |
| BRACHYURY RV | GCAGGTGAGTTGTCAGAATAGGT |
| GATA4 FW | GTCATCTCACTACGGGCACA |
| GATA4 RV | CTTCAGGGCCGAGAGGAC |
| PAX6 FW | TGACAGAAGCTGTGACAACCA |
| PAX6 RV | GCTCGAATATGGGGCTCTGA |
| BTG2 FW | AGGCACTCACAGAGCACTAC |
| BTG2 RV | TGGGGTCCATCTTGTGGTTG |
| SOX2 FW | ATGGGTTCGGTGGTCAAGTC |
| SOX2 RV | CTGATCATGTCCCGGAGGTC |
| mSOX1 FW | CCCATGCACCGCTACGACAT |
| mSOX1 RV | CGCTCATGTAGCCCTGAGAGT |
| mB-ACTIN FW | TCCTTCTTGGGTATGGAATCCTG |
| mB-ACTIN RV | AGGTCTTTACGGATGTCAACG |
| mOCT4 FW | GAGACTTTGCAGCCTGAGGG |
| mOCT4 RV | CTTTCATGTCCTGGGACTCCTC |
| mNESTIN FW | GAGGCGCTGGAACAGAGATT |
| mNESTIN RV | CACAGCCAGCTGGAACTTTTC |
| mBRACHYURY FW | GGGTATTCCCAATGGGGGTG |
| mBRACHYURY RV | CGGTGGTTCCTTAGAGCTGG |
| mGATA4 FW | CTCCATGTCCCAGACATTCAGT |
| mGATA4 RV | GATGCATAGCCTTGTGGGGA |
| mPAX6 FW | CCGTGCGACATTTCCCGAAT |
| mPAX6 RV | ACACAACCGTTGGATACGTTTT |
| mBTG2 FW | ACGCACTGACCGATCATTACAA |
| mBTG2 RV | GGGGTCCATCTTGTGGTTGA |
| mGAPDH FW | TGGAGAAACCTGCCAAGTATGAT |
| mGAPDH RV | GGTCCTCAGTGTAGCCCAAG |
| mSOX2 FW | CAGCATGTCCTACTCGCAGC |
| mSOX2 RV | TGGAGTGGGAGGAAGAGGTAA |
