# Supplementary material for: Highly efficient methods to obtain homogeneous dorsal neural progenitor cells from human and mouse embryonic stem cells and induced pluripotent stem cells
Source: Stem Cell Res Ther. 2018 Mar 15;9:67. doi: 10.1186/s13287-018-0812-6 (PMC5856210; doi:10.1186/s13287-018-0812-6)
Supplement: Supplementary file 3 — is Table S3 presenting testing for statistically significant differences in PAX6 and SOX1 IHC staining between single BMP and double BMP/SMAD neural induction protocols. Significance testing for differences in NPC marker expression for each human cell line in the single BMP and double BMP/SMAD inhibition protocols. Student's unpaired T-test and F test for variance were performed. P-values are shown comparing the ICC quantification in Fig. 1c and Fig. 2c; p-values <0.05 were considered significant. (DOCX 14 kb) [file 13287_2018_812_MOESM3_ESM.docx]

**Table S3:** Testing for statistically significant differences in PAX6 and SOX1 IHC staining between single BMP and double BMP/SMAD neural induction protocols.

| **BMP inhibition vs. BMP/SMAD inhibition** | **Unpaired T-test** | | **F test (compare significance of variance)** | |
| --- | --- | --- | --- | --- |
| **Cell line** | **PAX6** | **SOX1** | **PAX6** | **SOX1** |
| **H1 (hESC)** | < 0.0001 **** | 0.0184 * | 0.4870 | 0.2215 |
| **YH10 (iPSC)** | 0.1327 | 0.9456 | 0.9008 | 0.7492 |
| **BJ4 (iPSC)** | 0.0042 ** | 0.0075 ** | 0.0234 * | 0.0001 *** |
| **1323-2 (iPSC)** | 0.1039 | 0.0094 ** | 0.1034 | 0.0837 |

Significance testing for differences in NPC marker expression for each human cell line in the single BMP and double BMP/SMAD inhibition protocols. Student’s unpaired T-test and F test for variance were performed. *P*-values are shown comparing the ICC quantification in **Fig 1C** and **Fig 2C**; *p*-values <0.05 were considered significant.
