# Supplementary figures and images for: Highly efficient methods to obtain homogeneous dorsal neural progenitor cells from human and mouse embryonic stem cells and induced pluripotent stem cells
Source: Stem Cell Res Ther. 2018 Mar 15;9:67. doi: 10.1186/s13287-018-0812-6 (PMC5856210; doi:10.1186/s13287-018-0812-6)

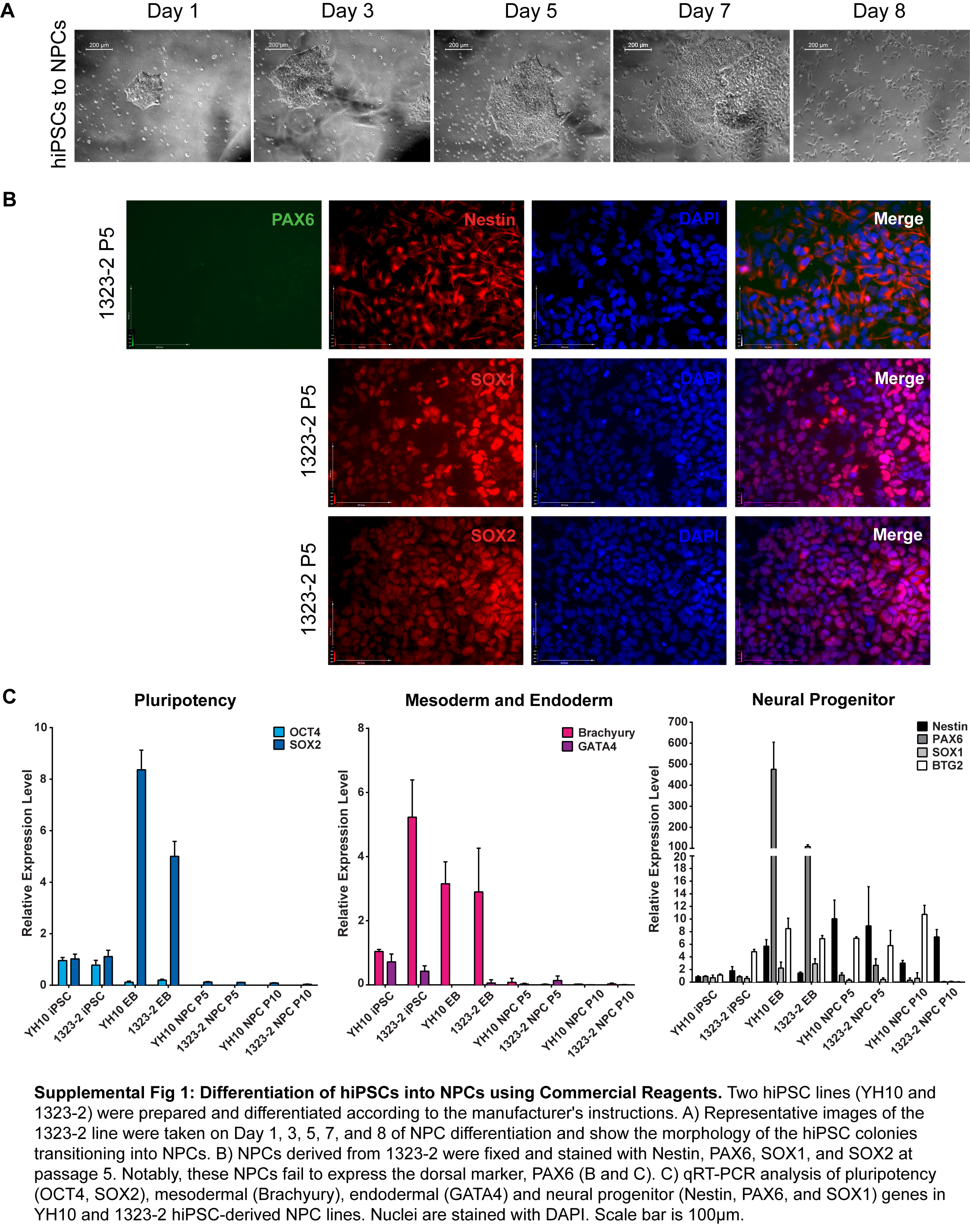

Supplement: Supplementary file 4 — is Figure S1 showing differentiation of hiPSCs into NPCs using commercial reagents. Two hiPSC lines (YH10 and 1323–2) were prepared and differentiated according to the manufacturer’s instructions. (A) Representative images of 1323–2 line taken on days 1, 3, 5, 7, and 8 of NPC differentiation showing morphology of hiPSC colonies transitioning into NPCs. (B) NPCs derived from 1323–2 fixed and stained with Nestin, PAX6, SOX1, and SOX2 at passage 5. Notably, these NPCs fail to express dorsal marker PAX6 (B, C). (C) qRT-PCR analysis of pluripotency (OCT4, SOX2), mesodermal (Brachyury), endodermal (GATA4), and neural progenitor (Nestin, PAX6, and SOX1) genes in YH10 and 1323–2 hiPSC-derived NPC lines. Nuclei stained with DAPI. Scale bar 100 μm. (TIFF 9638 kb) [file 13287_2018_812_MOESM4_ESM.tif]

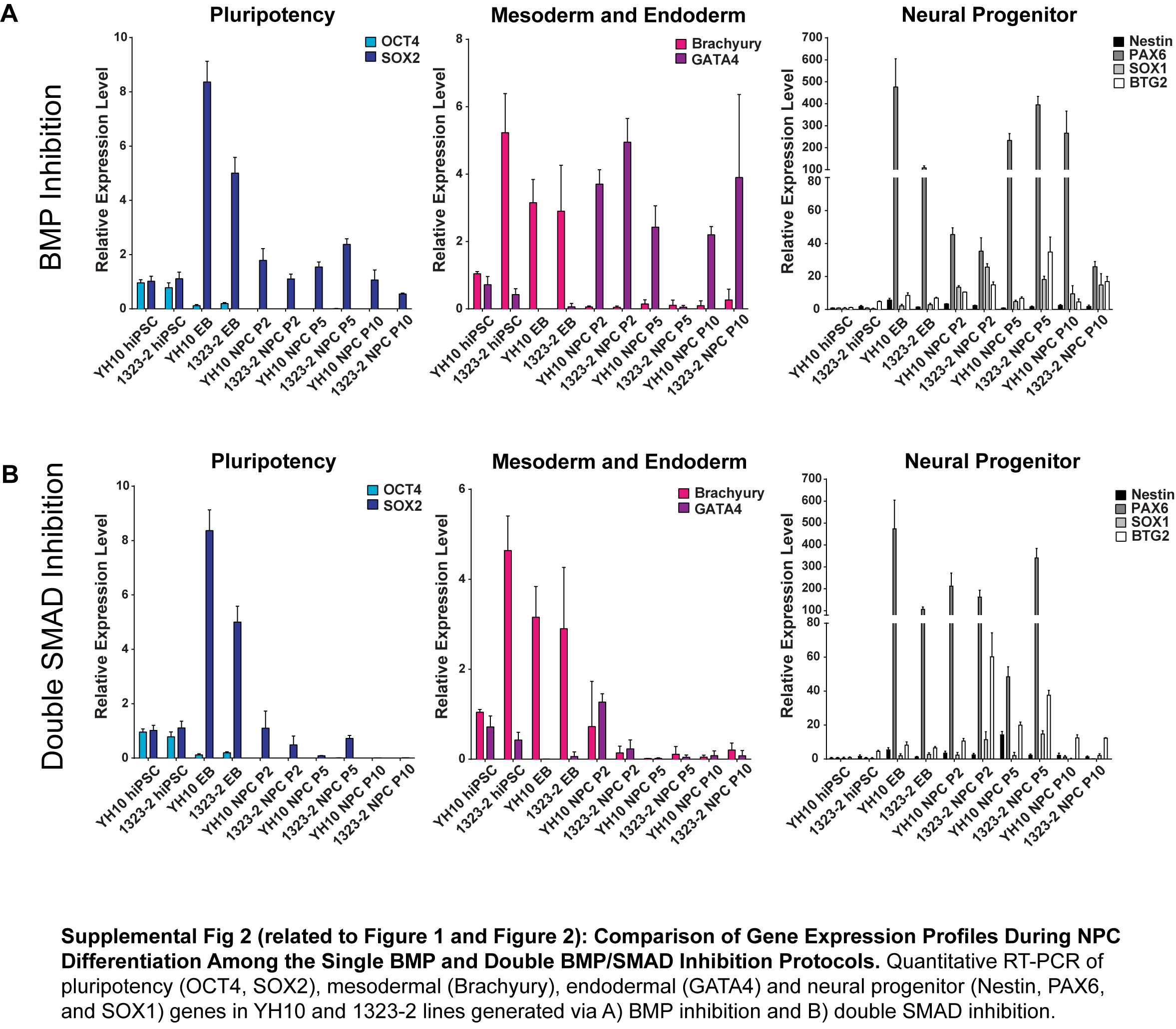

Supplement: Supplementary file 5 — is Figure S2 (related to Figs. 1 and 2) showing comparison of gene expression profiles during NPC differentiation among the single BMP and double BMP/SMAD inhibition protocols. (A, B) qRT-PCR of pluripotency (OCT4, SOX2), mesodermal (Brachyury), endodermal (GATA4), and neural progenitor (Nestin, PAX6, and SOX1) genes in YH10 and 1323–2 lines generated via (A) BMP inhibition and (B) double SMAD inhibition. (TIFF 2381 kb) [file 13287_2018_812_MOESM5_ESM.tif]

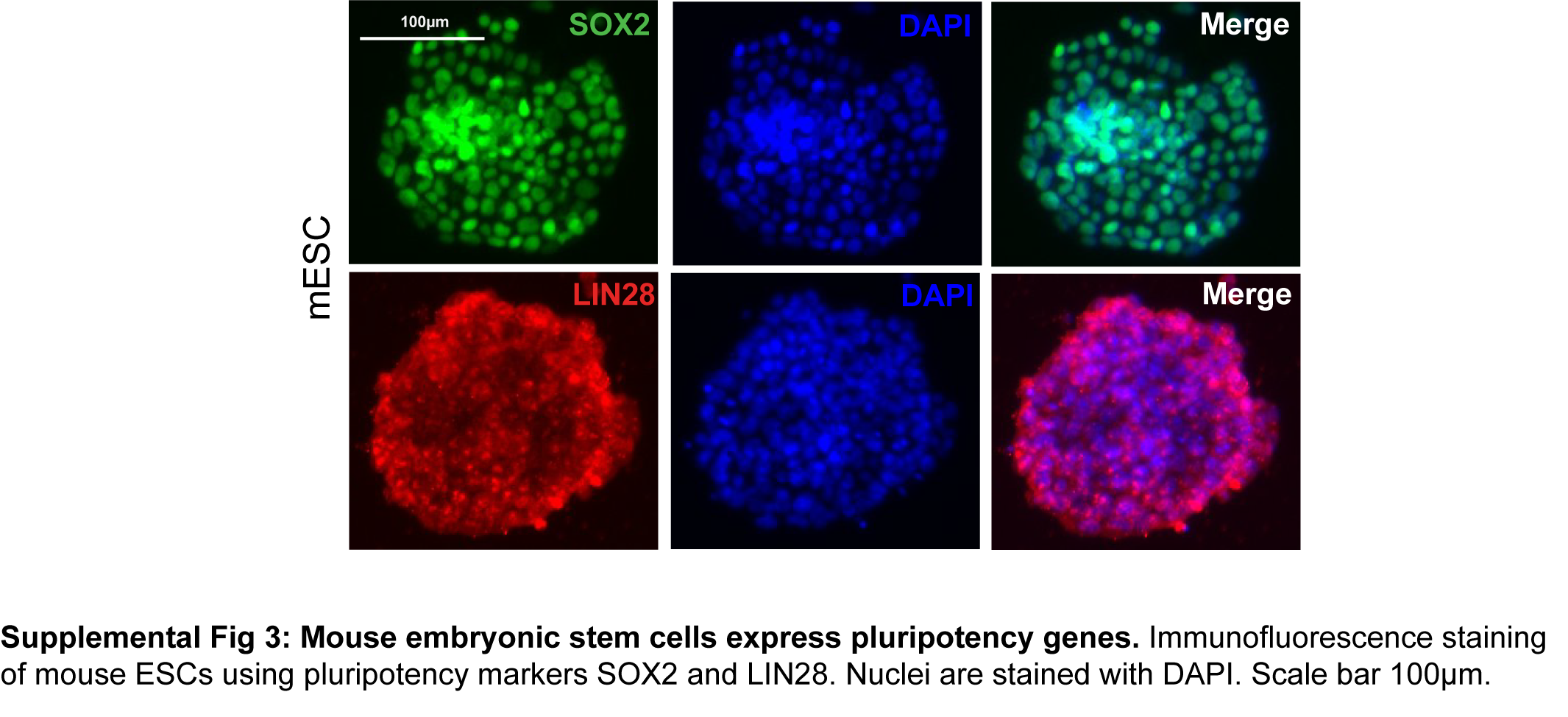

Supplement: Supplementary file 6 — is Figure S3 showing mESCs express pluripotency genes. Immunofluorescence staining of mESCs using pluripotency markers SOX2 and LIN28. Nuclei stained with DAPI. Scale bar 100 μm. (TIFF 3029 kb) [file 13287_2018_812_MOESM6_ESM.tif]

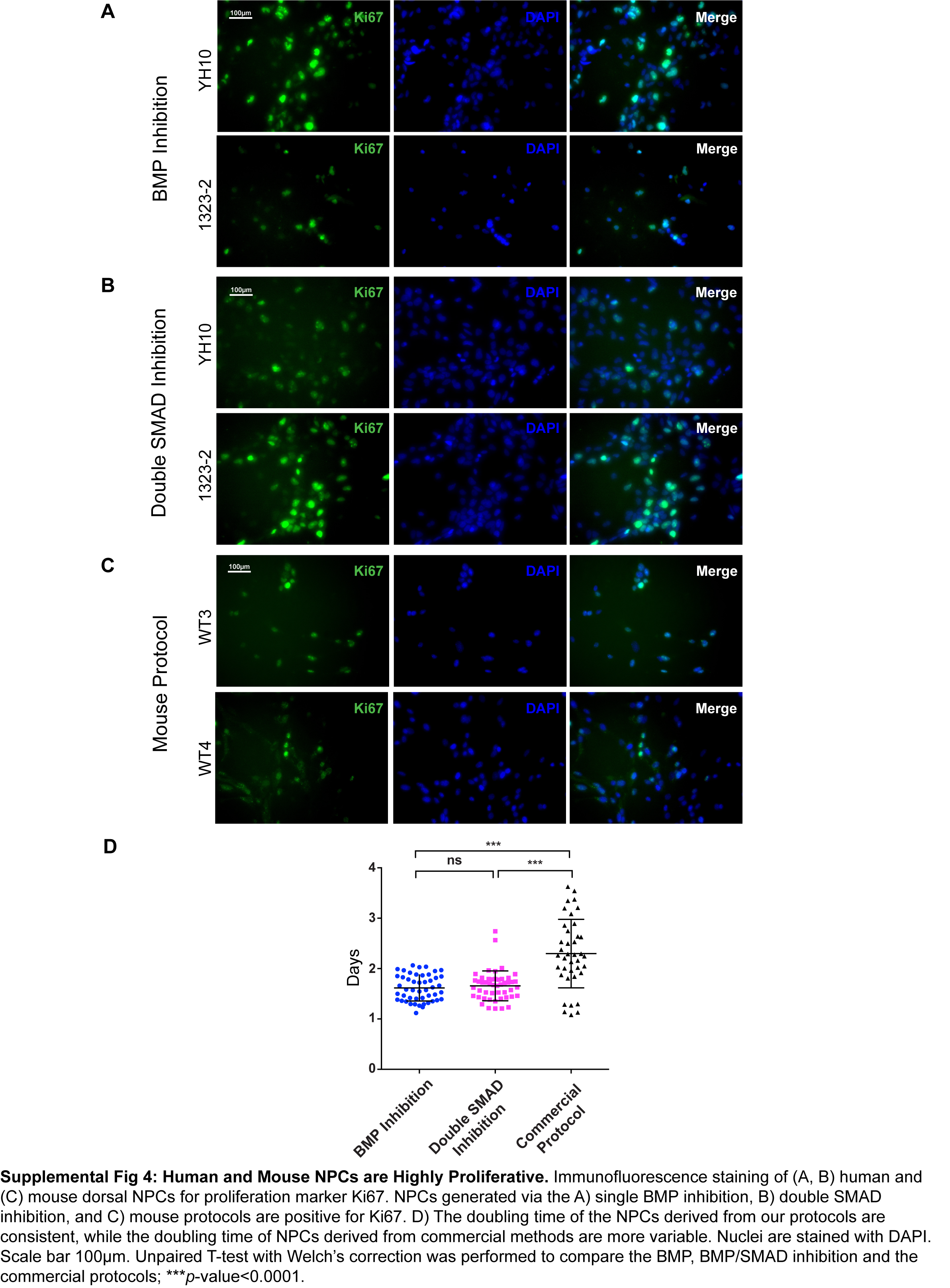

Supplement: Supplementary file 7 — is Figure S4 showing human and mouse NPCs are highly proliferative. Immunofluorescence staining of (A, B) human and (C) mouse dorsal NPCs for proliferation marker Ki67. NPCs generated via (A) single BMP inhibition, (B) double SMAD inhibition, and (C) mouse protocols are positive for Ki67. (D) Doubling time of NPCs derived from our protocols are consistent, while doubling time of NPCs derived from commercial methods are more variable. Nuclei stained with DAPI. Scale bar 100 μm. Unpaired t test with Welch’s correction was performed to compare BMP inhibition, BMP/SMAD inhibition, and commercial protocols. ***p < 0.0001. (TIFF 7781 kb) [file 13287_2018_812_MOESM7_ESM.tif]

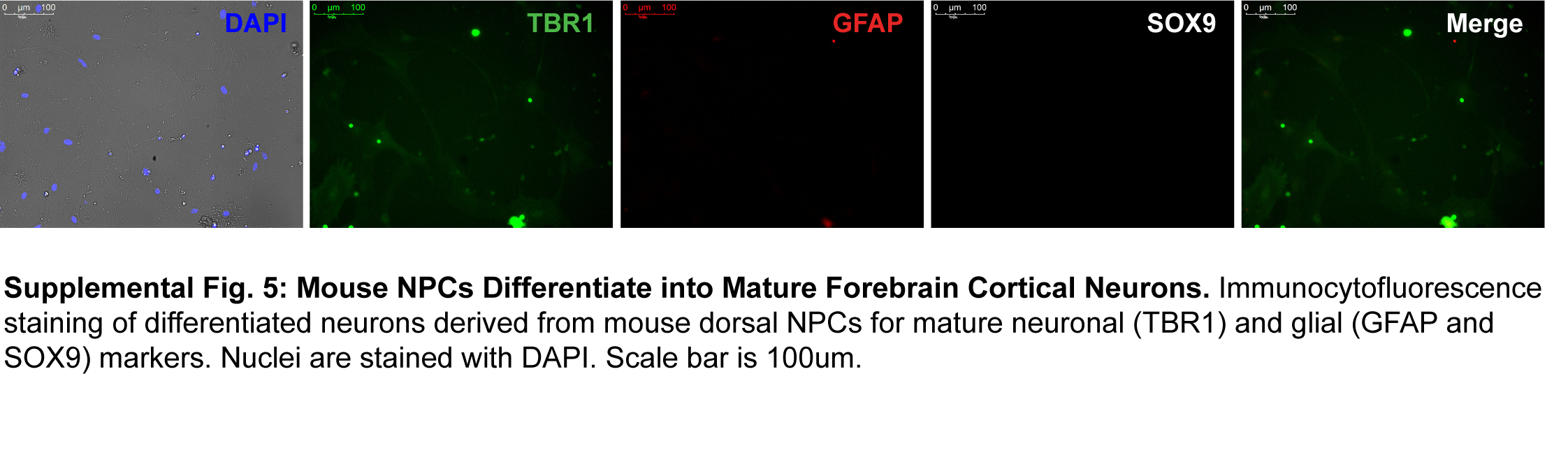

Supplement: Supplementary file 8 — is Figure S5 showing mouse NPCs differentiate into mature forebrain cortical neurons. Immunocytofluorescence staining of differentiated neurons derived from mouse dorsal NPCs for mature neuronal (TBR1) and glial (GFAP and SOX9) markers. Nuclei stained with DAPI. Scale bar 100 μm. (TIFF 2348 kb) [file 13287_2018_812_MOESM8_ESM.tif]
